# The effect of bubble size on the efficiency and economics of harvesting microalgae by foam flotation

**DOI:** 10.1007/s10811-014-0384-5

**Published:** 2014-08-07

**Authors:** Thea Coward, Jonathan G. M. Lee, Gary S. Caldwell

**Affiliations:** 1 School of Chemical Engineering and Advanced Materials, Merz Court, Newcastle University, Newcastle upon Tyne, NE1 7RU England UK; 2 School of Marine Science and Technology, Ridley Building, Newcastle University, Newcastle upon Tyne, NE1 7RU England UK

**Keywords:** *Chlorella*, Biomass, Harvesting, Algae, Biofuel

## Abstract

The effect of bubble size and rise velocity on the efficiency of a foam flotation microalgae harvesting unit was determined. Three sparger and input airflow combinations were used: (1) limewood sparger with constant airflow, (2) ceramic flat plate sparger with constant airflow and (3) ceramic flat plate sparger with an oscillating airflow. The ceramic sparger with oscillating flow generated the smallest bubbles within the liquid pool and the largest bubbles within the foam phase. This delivered the highest levels of biomass recovery due to enhanced bubble-algae collision and attachment efficiencies. The smaller bubbles generated by the ceramic sparger under constant or oscillating airflow had significantly faster rise velocities when compared to the larger bubbles produced by the limewood spargers. The faster velocities of the smaller bubbles were due to momentum transfer to the liquid phase. Analyses of the harvest economics revealed that the ceramic flat plate sparger with an oscillating airflow delivered the best overall cost-benefit relationship.

## Introduction

Microalgae have considerable potential as a feedstock for the scalable, affordable and sustainable production of a wide range of bioproducts (Demirbas and Fatih Demirbas 2011; Yuan et al. 2011; Borowitzka 2013). Despite extensive research, bulk commodities derived from microalgae, such as biofuels, are not yet commercially viable (Borowitzka 2013). The harvesting and dewatering of large volumes of microalgae biomass remains one of several significant processing bottlenecks (Pahl et al. 2013; Chisti 2013). Significant research effort has focussed on developing harvesting technologies that improve biomass recovery; however, the majority of harvesting methods are not sufficiently cost effective (Coward et al. 2013; Sharma et al. 2013). Of the current technologies on offer, foam flotation—a process involving liquid and foam phases to separate surface-active particles from water—is regarded as among the more efficient methods to remove algae from suspension (Liu et al. 1999; Wiley et al. 2009; Coward et al. 2013, 2014; Hanotu et al. 2013; Sharma et al. 2013).

To ensure efficient harvesting by foam flotation, it is considered necessary to modify the hydrophilic interface of the microalgae cell (Garg et al. 2012). Cationic surfactants are commonly used to increase the hydrophobicity of the negatively charged cell when they are adsorbed on the cell surface. When combined with a low-pressure sparger generating bubbles within the liquid pool, the surfactant will also generate stable foam (Chen et al. 1998; Liu et al. 1999; Hanotu et al. 2012; Coward et al. 2013, 2014). Bubble size has been highlighted as one of the most important factors affecting the performance of foam flotation (Du et al. 2003; Wong et al. 2001). Bubbles perform a range of functions within the process. Within the liquid pool, bubble motion facilitates mixing and therefore increases the likelihood of algae-bubble interaction (Wong et al. 2001), bubbles provide the interface for the attachment of the microalgae cell, and bubbles transport the attached microalgae towards the foam-liquid interface. Within the foam phase, bubble coalescence encourages liquid drainage and provides an internal reflux that increases the concentration factor of the harvest (Du et al. 2001; Wong et al. 2001). Concentration factor is defined in Eq. 1 (Coward et al. 2013):

1 $$ CF=\frac{\left({\mathrm{cells}\ \mathrm{cm}}^{-3}\right)\mathrm{suspension}}{\left({\mathrm{cells}\ \mathrm{cm}}^{-3}\right)\mathrm{harvest}} $$

Within the liquid pool, it is important to optimise the interaction, attachment and stability efficiencies between the bubble and microalga (Derjaguin and Dukhin 1993). An effective way to improve these variables is to reduce bubble size, thereby increasing the surface area per unit volume and enhancing the likelihood of bubble-algae interaction (de Rijk et al. 1994; Hanotu et al. 2012, 2013; Pahl et al. 2013). By converting a constant airflow into an oscillating airflow using fluidic oscillators based on the Coandă effect, Zimmerman and co-workers have developed an energy-efficient method for the generation of micro bubbles of a given size (Zimmerman et al. 2008, 2009, 2011; Tesar and Bandalusena 2011; Al-Mashhadani et al. 2012; Hanotu et al. 2012, 2013). When the airflow is constant, an anchoring force attaches the bubble to the exit pore of the sparger. To overcome this force, the bubble must grow until the point when the buoyant force exceeds the anchoring force. This generates bubbles that are significantly larger than the exit pore of the sparger (Zimmerman et al. 2008). During oscillating flow, a pulse is created which provides a lifting force that enables the bubble to break free from the anchoring force at an earlier stage, thereby producing a significantly smaller bubble (Hanotu et al. 2012). Hanotu et al. (2012) generated bubbles with a mean radius of 86 μm using oscillatory flow and a stainless steel baffle distributor sparger. The bubbles effectively separated *Dunaliella salina* recovering up to 99.5 % of the biomass. However, Hanotu et al. (2012) used dispersed air flotation, wherein the algae cells were recovered from the top of the liquid interface without the formation of a foam phase. Within a rising foam phase of foam flotation, smaller bubbles are closely linked to a decrease in foam drainage. This leads to a dilute harvest (Du et al. 2001; Wong et al. 2001; Li et al. 2011; Coward et al. 2013) necessitating further biomass processing with associated increased energy requirements.

The dispersed air flotation work of Hanotu et al. (2012, 2013) also required high dosages of metallic coagulants (150 mg L^−1^ ferric chloride) to increase the hydrophobicity of the microalgae. Previous work using a freshwater *Chlorella* sp. demonstrated effective foam flotation harvesting using as little as 10 mg L^−1^ of the cationic surfactant cetyltrimethylammonium bromide (CTAB) (Coward et al. 2013, 2014). Combining CTAB-assisted foam flotation with the miniaturisation of bubbles within the liquid pool could deliver an attractive low-cost harvesting method that would require significantly lower rates of chemical addition compared with dispersed air flotation.

A broad distribution of bubble sizes affects the overall foam flotation process. It is therefore important to report bubble size distributions in both the liquid pool and the foam phase. To what extent the method of bubble production affects bubble size in the foam phase, and consequently the harvest economics, has yet to be reported. Conceptually, the ideal separation would occur when small bubbles are produced in the liquid pool which then coalesce forming larger bubbles in the foam phase (Li et al. 2011). This study thus investigated the effect of the size distribution and velocity of bubbles generated by three contrasting sparger and airflow setups on the biomass concentration factor, biomass recovery and dewatering process economics of *Chlorella* sp. harvested by CTAB-assisted foam flotation.

## Materials and methods

### Sparger setups

**Fig. 1:**
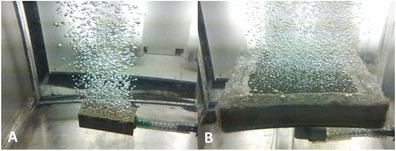
Bubble clouds generated using two sparger setups used in foam flotation. **a** A limewood sparger with constant flow produced bubbles with a Sauter mean diameter of 1,229 ± 155 μm; **b** a ceramic flat plate sparger with an oscillating flow produced bubbles with a Sauter mean diameter of 622 ± 59 μm

**Fig. 2:**
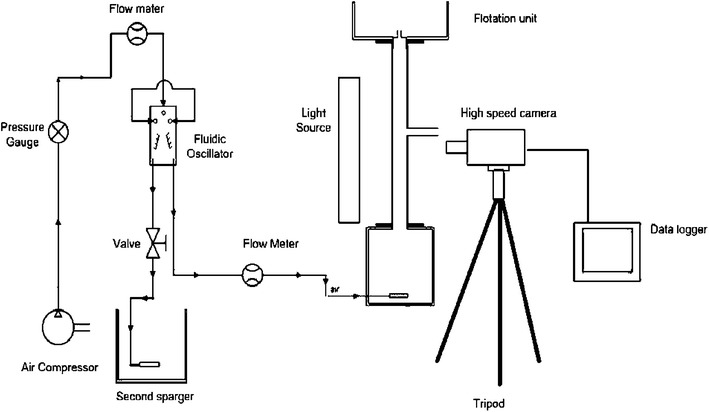
Experimental setup used to determine bubble size distribution and velocity; in this instance, the setup is for use with an oscillating flow. For a constant flow, the air went from the compressor to the pressure gauge, through one flow meter and into the bottom of the flotation unit

Three sparger and airflow setups were used to generate differing bubble sizes: a limewood sparger with constant airflow (LCF), a ceramic flat plate sparger with constant airflow (CCF) and a ceramic flat plate sparger with fluidic oscillator to create an oscillating airflow (COF). The limewood sparger had dimensions of 2 cm × 2 cm × 5 cm in height, width and length, respectively, with a mean pore diameter of 35.0 μm (Yang et al. 2004). The ceramic flat plate sparger had dimensions of 2 cm × 11 cm × 11 cm in height, width and length, respectively, with a mean pore diameter of 20.0 μm (Al-Mashhadani et al. 2012). The liquid pool was held within the flotation harvester’s base tank, which had inner dimensions of 22 cm × 22 cm × 21.1 cm, with a 10.2 L working culture volume (Coward et al. 2013). The spargers were located centrally within the base tank. For the limewood sparger, the air entered 2 cm above the base of the liquid pool. The ceramic flat plate sparger had an inbuilt elbow necessary to distribute the air evenly across the plate; this resulted in the sparger being located 6 cm above the base of the liquid pool (Fig. 1). During all experimental trials, the liquid pool height was 20.7 cm. The current study used a foam column height of 50 cm and 4.6 cm internal diameter. A steady airflow rate of 3,700 L h^−1^ was supplied to the fluidic oscillator to induce an oscillating flow. For all sparger setups, the airflow entering the foam flotation unit was regulated to 100 L h^−1^ with the use of a flow meter. To achieve this for COF, a portion of the air was bled off and channelled to another sparger (Hanotu et al. 2014), and a flow meter added just before the airflow entered the base chamber (Fig. 2). To determine the effect of each sparger setup on foam flotation harvesting, 2 L of concentrated microalgae culture was added to 7.5 L of tap water plus 500 mL of CTAB solution to give a cell density of 4.1 × 10^7^ ± 9.6 × 10^6^ cell mL^−1^ at a CTAB concentration of 10 mg L^−1^.

### Bubble analysis

Bubble size, distribution and velocity were determined optically using an Olympus i-SPEED 2 camera (800 × 600 pixel image quality) coupled to a Computar 18–80 lens (no. 5001568) with a focal distance of 22.8 ± 0.23 cm. The flotation unit was backlit using ‘Nebula 4’ hydroponic plant lights, which were each fitted with four vertically mounted Philips PL-L 4P 55 W florescent lamps. A minimum of 200 images were captured at 1,000 frames per second for each sparger setup. Images were captured at 5-min intervals over a 30-min period to obtain information on how bubble size changes within the foam phase as the surfactant becomes exhausted. Images were converted to JPEG files using the Olympus *i-SPEED* Control Pro software and analysed using ImageJ software (National Institutes of Health, Bethesda, MD, USA). The circumferences of 200–300 bubbles were measured and then converted to diameter. The bubble velocity within the liquid pool and the foam phase was determined from the images by recording distance moved over a known time frame. Each reported bubble velocity value is a mean of 40 measurements.

A ruler was placed on the outside wall of the base chamber and foam column to calibrate all images by conversion to pixel number. To image the liquid pool, the camera was set up 4 cm above the bottom of the chamber; to image the midpoint of the foam column, the camera was set up 46 cm above the bottom of the chamber. Due to turbidity of the liquid pool caused by the microalgae, bubble characterisation was performed prior to harvesting with the addition of 10 mg L^−1^ of CTAB in the culture chamber. To minimise any magnification artefacts, only bubbles nearest the harvester walls were analysed (Wong et al. 2001). A distortion test was conducted by placing 10-mm glass spheres into the column; they were surrounded by a CTAB solution of the same strength as that used in the harvesting experiments. The diameter of the spheres was measured using the same optical setup as previously described to measure the bubble diameter in the foam. The distortion caused by the walls of the tube was found to be 5 % of the real diameter and was considered small enough to be discounted. A schematic representation of the experimental setup is shown in Fig. 2.

### Microalgae separation

The microalgae growth conditions are described elsewhere (Coward et al. 2013). All harvests were conducted as batch under the following conditions: foam column height of 0.5 m, airflow rate of 100 L h^−1^ and 30-min batch run time. Each harvest was conducted with four replicates at room temperature. The foam was collected after each harvest and allowed to collapse. The volume of liquid was measured and samples were taken from the harvested material. The cell density was determined using an improved Neubauer hemocytometer. The concentration factor and biomass recovery of each harvest were calculated using Eq. 1 (Coward et al. 2013).

### Harvest economics

Small changes within a flotation harvest system, such as operational air pressure, can dramatically affect the work required for an air compressor, therefore affecting the energy consumption and economics of the harvest system. An economic assessment was conducted to compare the efficiency and energy consumption of each sparger setup to harvest 1,000 L of a *Chlorella* sp. Comparisons were made of the associated chemical costs for foam flotation using 10 mg L^−1^ CTAB (Coward et al. 2013, 2014), and flocculation and dispersed air flotation requiring the culture to be adjusted to pH 5 and the addition 150 mg L^−1^ of ferric chloride (Hanotu et al. 2012, 2013). The total energy consumed (kWh m^−3^), current energy costs (US$ kWh^−1^), chemical dosage requirement (mg L^−1^), chemical costs (US$ kg^−1^) and the cost of pH adjustment were taken into account to determine the total cost of harvesting. The specific work of an air compressor was calculated using Eq. 2:

2 $$ W=\frac{n_{air} R{T}_1}{\eta_{isen}\left(\gamma -1\right)}\left[{\left(\frac{P_2}{P_1}\right)}^{\frac{\gamma -1}{\gamma}}-1\right] $$

where *W* is the work in J mol^−1^; n_air_ is the molar flow rate of air; *R* is the gas constant, assumed to be 8.314 J mol^−1^ K^−1^; *T*_1_ is the temperature in kelvins, assumed to be 293 K; *P* is pressure; *γ* is the specific heat ratio of air, assumed to be 1.4; and η_isen_ is the isentropic efficiency of the air compressor, assumed to be 0.5. The pressure required for the COF sparger was measured with a pressure gauge (WIKAI, 0–2.5 bar); for the LCF and CCF spargers, a manometer was used as the pressure requirements for the spargers were below the lower limit that could be read on the pressure gauge. The manometer was mercury-filled, and the height differential was measured when connected to a working harvesting unit. Gauge pressure was calculated using Eq. 3:

3 $$ \varDelta P= P-{P}_0=\rho gh $$

where *P*_0_ is atmospheric pressure, *ρ* is the fluid density of the reference liquid, *g* is the acceleration of gravity (9.81 m s^−2^), and *h* is the height of the fluid in the column.

### Statistical analysis

Data were tested for normality using an Anderson-Darling Normality Test, comparing the empirical cumulative distribution function of the sample data with the distribution that would be expected if the data were normally distributed. Normally distributed data were compared using an analysis of variance (ANOVA) test. Data that were not normally distributed were analysed using the Mood’s median nonparametric test.

## Results

### Bubble size distribution within the liquid pool and foam phase

**Fig. 3:**
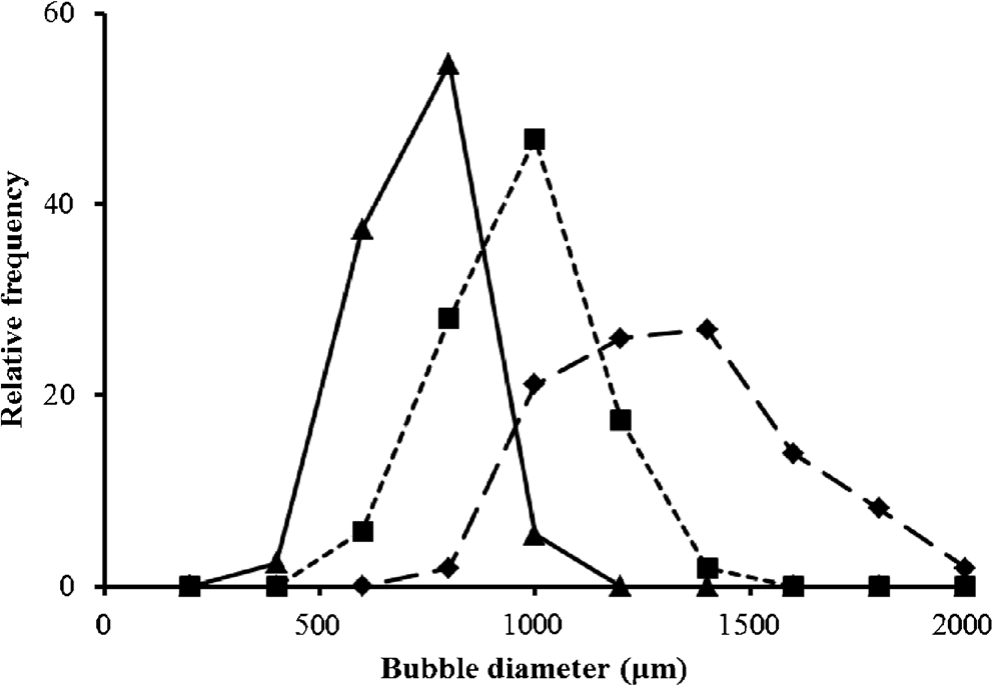
Bubble size distribution within the liquid pool after 5 min of harvester run time. *Long dashed line* with *diamond marker* = limewood sparger with constant flow (LCF); *short dashed line* with *square marker* = ceramic flat plate sparger with constant flow (CCF); and *solid line* with *triangular marker* = ceramic flat plate sparger with oscillating flow (COF)

The bubble size distribution within the liquid pool under constant and oscillating airflow conditions is presented in Fig. 3. Under constant flow, the limewood sparger generated a wide bubble size distribution ranging from 673 to 1,955 μm, with a Sauter mean diameter of 1,229 μm. The distribution peak exhibited a negative skew and highlights a dominance of bubbles within the size range of 1,200–1,400 μm (representing only 26.9 % of the distribution). The ceramic flat plate sparger with constant airflow generated significantly smaller bubbles with a more compact distribution pattern relative to the limewood sparger (*p* = <0.001) ranging from 437 to 1,343 μm, with a Sauter mean diameter of 859 μm. The more confined bubble distribution had a high frequency of bubbles between 800 and 1,000 μm, representing 46.9 % of the distribution.

Changing from a constant to an oscillated airflow further significantly reduced bubble size (*p* = <0.001) producing a distribution pattern exhibiting a positive skew (Fig. 3). Bubbles were generated in the size range of 348–981 μm, with a Sauter mean diameter of 622 μm. The dominant size range was between 600 and 800 μm, representing 54.7 % of the distribution.

**Fig. 4:**
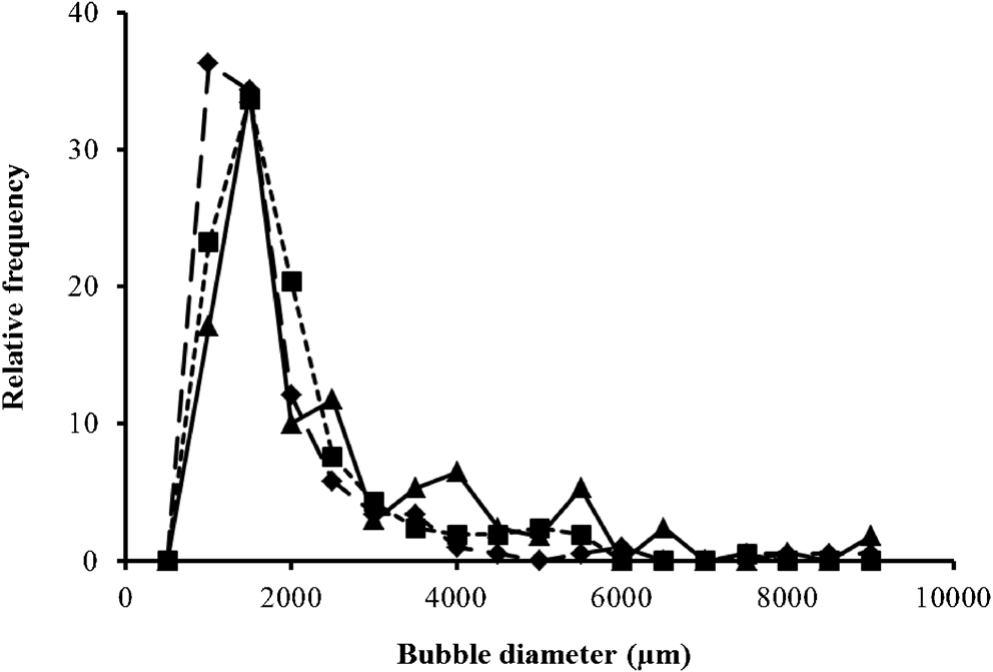
Bubble size distribution within the foam phase after 5 min of harvester run time. *Long dashed line* with *diamond marker* = limewood sparger with constant flow (LCF); *short dashed line* with *square marker* = ceramic flat plate sparger with constant flow (CCF); and *solid line* with *triangular marker* = ceramic flat plate sparger with oscillating flow (COF)

Within the foam phase, bubble size measurements taken at 5-min intervals revealed that mean bubble size produced by the LCF setup increased significantly (*p* = <0.001) from 1,413 to 2,485 μm. Significant increases (data not shown) were also observed for both the CCF (*p* = 0.047) and COF setups (*p* = 0.007). A more detailed analysis was conducted on foam phase bubbles after 5 min into the flotation run time as previous studies have demonstrated that up to 85 % of microalgae cells are removed within the first 5 min (Chen et al. 1998; Liu et al. 1999). Within the foam phase, the bubble distribution for all sparger setups peaked between 1,000 and 1,500 μm, with similar bubble frequencies of 34.3, 33.6 and 34.1 % for the LCF, CCF and COF setups respectively (Fig. 4). However, the COF had a significantly different size distribution to the constant flow sparger setups (*p* = <0.001), with the primary peak followed by several smaller peaks. This is reflected in the mean bubble diameters of 1,481, 1,708 and 2,305 μm for the LCF, CCF and COF setups, respectively.

The mean bubble sizes within the foam phase produced by constant flow methods were very similar (*p* = 0.998); however, the foam produced by the oscillating flow produced significantly larger bubbles (*p* = <0.001).

### Bubble rise velocity

**Fig. 5:**
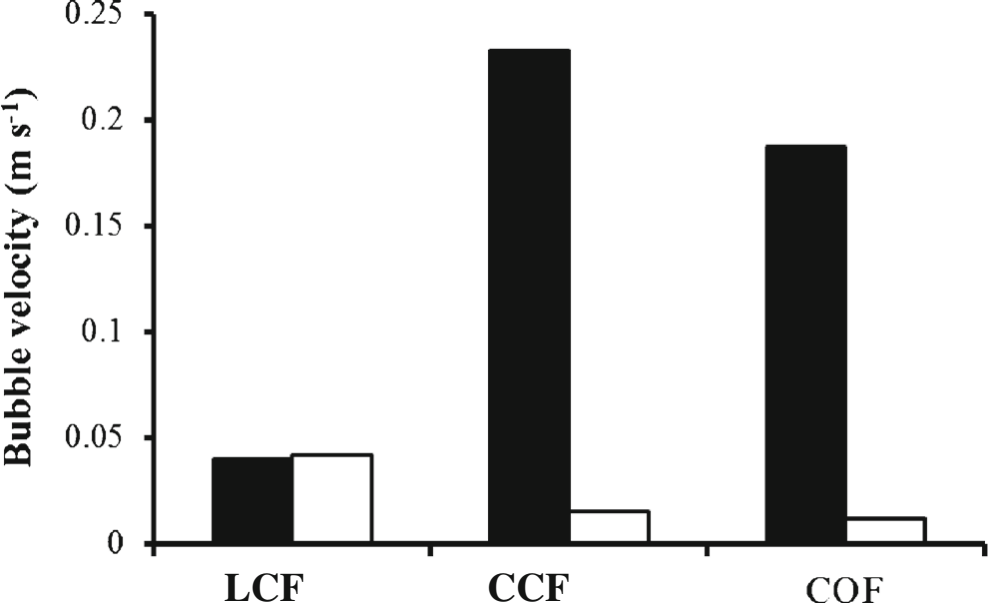
Bubble velocity within the liquid pool (*black fill*) and foam phase (*white fill*) after 5 min of harvester run time. *LCF* = limewood sparger with constant flow, *CCF* = ceramic flat plate sparger with constant flow, and *COF* = ceramic flat plate sparger with oscillating flow

Within the liquid pool (Fig. 5), the bubbles produced by the LCF had a rise velocity of 0.04 m s^−1^ which was significantly slower when compared with bubbles produced by the CCF and COF sparger setups (*p* value = <0.001), which had rise velocities of 0.23 and 0.19 m s^−1^ respectively. Figure 5 shows the rise velocity of bubbles within the foam phase 5 min into the run time. The foam produced by the LCF setup had a significantly faster (*p* = <0.001) rise velocity (peaking at 0.042 m s^−1^) compared with the foam produced by the CCF (peaking at 0.016 m s^−1^) and COF (peaking at 0.012 m s^−1^) setups. This resulted in no significant difference between the bubble velocity within the liquid pool and the foam phase for the LCF setup (*p* = 0.581). The rise velocity for the LCF setup changed substantially over the course of the run period; for example, the peak velocity at 5 min was 2.7 times faster than the mean rise velocity of the entire run. In contrast, the rise velocity of the CCF and COF setups remained relatively constant (0.014 ± 0.0014 and 0.012 ± 0.0007 m s^−1^ respectively).

### Algal harvest

**Fig. 6:**
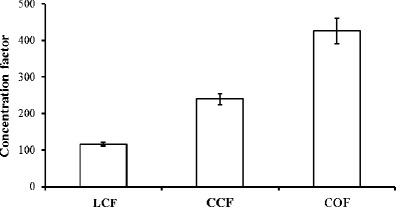
The harvest concentration factor (defined in Eq. 1) gained using each of three different sparger/airflow setups. *LCF* = limewood sparger with constant flow, *CCF* = ceramic flat plate sparger with constant flow, and *COF* = ceramic flat plate sparger with oscillating flow

**Fig. 7:**
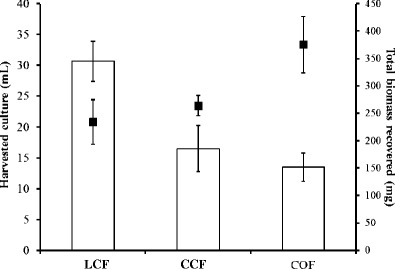
The volume of harvested culture (*columns*) relative to the biomass recovered (*filled squares*) for each of three different sparger/airflow setups. *LCF* = limewood sparger with constant flow, *CCF* = ceramic flat plate sparger with constant flow, and *COF* = ceramic flat plate sparger with oscillating flow. These equate to biomass concentrations within the harvester collection cup of 7.64 g L^−1^ for LCF, 16 g L^−1^ for CCF, and 27.8 g L^−1^ for COF

Sparger type and setup had a major influence on the harvest concentration factor and biomass recovery. Figure 6 shows that the COF setup achieved high concentration factors of 427 ± 35, which approximates to performance improvements of 78 and 267 % relative to the CCF and LCF setups, respectively. Figure 7 shows the volume of culture that needs to be processed relative to the biomass recovered; this is an important consideration that significantly impacts the economics of further processing. The COF setup achieved the most favourable biomass to culture volume ratio within the batch system leaving only 13.5 mL of residual culture volume from the recovery of 376 mg of microalgae biomass (equivalent to a biomass concentration of 27.8 g L^−1^) compared with 30.7 mL with the recovery of 234 mg of biomass for the LCF setup (equivalent to a biomass concentration of 7.6 g L^−1^).

### Harvest economics

Table 1 shows the air pressure requirements for each sparger and its influence on the energy consumption of flotation systems. The efficiency of an air compressor is never 100 % due to energy lost as heat; therefore, 50 % efficiency was assumed for the foam flotation harvester. The LCF and CCF setups had similar energy consumptions of 0.015 and 0.019 kWh m^−3^ respectively, due to the low operational pressure of 0.04 and 0.05 bar for the LCF and CCF setups respectively. Changing from a constant to an oscillated airflow increased the energy consumption by up to seven times that of LCF to 0.105 kWh m^−3^, and operational pressures of 0.3 bar.

**Table 1:** The energy consumption of an air compressor, assuming efficiencies of 50 %, delivering air to three sparger setups used in foam flotation: (1) limewood with constant flow, LCF; (2) ceramic flat plate with constant flow, CCF; and (3) ceramic flat plate with oscillating flow, COF

Flotation setup	Airflow (L h^−1^)	Pressure (bar)	Energy consumption of air compressor, 50 % efficiency (kWh m^−3^)
LCF	100	0.04	0.015
CCF	100	0.05	0.019
COF	100	0.3	0.105

Table 2 demonstrates that the cost of foam flotation using LCF, CCF or COF setups are similar with all system setups harvesting 1,000 L of algae at a calculated cost of US$0.0731, US$0.0734 and US$0.0791 respectively. Combining dispersed air flotation with fluidic oscillators, chemical dosing and pH adjustments as demonstrated by Hanotu et al. (2012, 2013) to induce flocculation followed by flotation results in a costly harvesting process (minimum of US$0.915 per 1,000 L). The foam flotation system using the COF setup, dosing 10 mg L^−1^ CTAB, in comparison had a calculated total harvesting cost of US$0.08 per 1,000 L, a substantial improvement on the dispersed air flotation method.

**Table 2:** The predicted cost of harvesting 1,000 L of algae suspension using different flotation harvesting techniques

	LCF	CCF	COF	Dispersed air flotation + flocculants
Total energy consumed (kWh)	0.015	0.019	0.105	0.105
Energy cost (US$ per kWh) ^b^	0.0010	0.0013	0.0070	0.0070^a^
Chemical additive (g)	10 (CTAB)	10 (CTAB)	10 (CTAB)	150 (FeCl_3_)
Chemical cost (US$/kg^−1^)	7.21^c^	7.21^c^	7.21^c^	0.4^d^
				52.76^e^
Cost of chemical per 1,000 L	0.0721	0.0721	0.0721	0.06^d^
				7.914^e^
Dosage for pH 5 adjustment	–	–	–	2.12 kg HNO_3_^f^
pH adjustment cost (US$)	–	–	–	0.848^g^
Cost to treat 1,000 L^−1^ (US$)	0.0731	0.0734	0.0791	0.915^d^
				8.769^e^

Cost factors are derived from air compressor energy consumption and chemical additives. Cost is given as United States of America dollars (US$). Data for dispersed air flotation + flocculants is based on Hanotu et al. (2012, 2013). The dosage of CTAB is 10 and 150 mg L^−1^ for iron chloride. For the purposes of comparing with Hanotu et al. (2012), we include two costs for the costs for FeCl_3_: a bulk purchase price and the price quoted by Hanotu et al. (2013)

*LCF* limewood with constant flow, *CCF* ceramic flat plate with constant flow, *COF* ceramic flat plate with oscillating flow

^a^Energy consumption of dispersed air flotation unit was assumed to be the same as COF. Energy consumption calculated using Eq. 2

^b^Electricity prices were calculated from the data presented by the US Department of Energy based on the average cost of electricity to the US industrial sector as of May 2013—US$ 0.0667 per kilowatt hour (Hankey 2013)

^c^Cetyltrimethylammonium bromide (CTAB) costs based on data from Sharma et al. (2013): AU$ 8 kg^−1^ with an exchange rate of US$ 1 = AU$ 1.11

^d^Based on a bulk price of US$ 400 t^−1^ (www.alibaba.com)

^e^Cost per kilogram of FeCl_3_ calculated by Hanotu et al. (2012), with an exchange rate of US$1 = GBP £0.66

^f^Calculated by experimentation with 1 L of *Dunaliella salina* in seawater

^g^Based on a bulk price of US$ 400 t^−1^ for 0.7 N HNO_3_ (www.alibaba.com)

## Discussion

### Bubble size distribution within the liquid pool and foam phase

Determining the bubble size distribution in the foam by photographing the foam through a transparent column and using imaging processing software is a commonly used experimental technique (Stevenson 2006). However, Stevenson et al. (2010) have noted how notoriously problematic gas–liquid foam systems are to experiment upon, due to the difficulty in measuring the bubble size distribution within the bulk of the foam. Cheng and Lemlich (1983) determined that measurements at the column wall may not be representative of the situation within the bulk of the foam due to planar sampling bias, which discriminates against smaller bubbles, and the fact that smaller bubbles can wedge larger ones from the wall, which discriminates against larger bubbles. X-ray tomography and pulsed-field gradient nuclear magnetic resonance (PFG-NMR) have been highlighted as techniques that will improve measuring of bubble size distribution in foam; unfortunately, neither of these techniques are readily available (Stevenson and Li 2012). However, Stevenson (2006) has stated that in the absence of these techniques, the experimentalist can assume that one source of error cancels the other.

Within the liquid pool, each sparger setup produced significantly different Sauter mean diameters and bubble size distributions. The difference was primarily due to the reduced exit pore size in the limewood and ceramic spargers (20 vs 35 μm, respectively). Overall, the bubbles produced using the oscillating airflow were of a more uniform size compared with a constant flow. This was due to regular detachment of the bubble from the exit pore as a consequence of the pulsed lifting force that reduced coalescence within the liquid pool (Hanotu et al. 2012).

Measurements taken at 5-min intervals across the 30-min harvesting period revealed that mean bubble size produced by all sparger setups increased significantly in the foam phase. During harvesting, the foam phase had a higher surfactant concentration than the liquid pool due to liquid drainage as the foam travels up the column, thereby enriching the surfactant at the gas–liquid interface. This work involved a batch system; therefore, bubble size increased with time due to surfactant exhaustion within the liquid pool. Bubbles within the foam phase were significantly larger and had a broader size range compared with bubbles within the liquid pool due to bubble coalescence during liquid drainage (Du et al. 2002).

The COF setup produced the best combination of bubble sizes, with the smallest mean bubble size within the liquid pool combined with the largest mean bubble size within the foam phase. The smaller bubbles within the liquid pool improved particle/bubble collision, attachment and stability efficiencies (Derjaguin and Dukhin 1993) increasing algae capture prior to reaching the liquid surface (de Rijk et al. 1994; Hanotu et al. 2012, 2013; Pahl et al. 2013). Within the rising foam phase, larger bubbles are correlated to an increase in foam drainage (Du et al. 2001; Wong et al. 2001; Li et al. 2011; Coward et al. 2013), increasing biomass concentration per litre harvested.

### Bubble rise velocity

The rise velocity of air bubbles within liquid dispersions is a key performance parameter as it describes the gas residence time and therefore affects the contact time for mass transfer across the interface (Kulkarni and Joshi 2005); however, it is commonly an unreported feature. For bubbles less than 300 μm in diameter, the rise velocity is dependent upon bubble size; larger bubbles have a greater buoyant-force-to-drag ratio than smaller bubbles. Therefore, reducing bubble size will reduce the rise velocity and increase the gas residence time within the liquid phase (Azgomi et al. 2007). However, between bubble sizes of 300 μm to 3 cm, Clift et al. (1978) predict a gradual reduction of the terminal rise bubble velocity with increasing bubble diameter. When considering the bubble size data produced for all three sparger setups, it is apparent that these data are roughly in agreement with this theory. The larger bubble diameters produced by LCF have an ellipsoidal shape, which are more prone to oscillations as they rise through the liquid phase, thereby reducing the rise velocity (Clift et al. 1978; Chisti 1989; Jamialahmadi et al. 1994). The higher velocities of the bubbles from the ceramic sparger can be explained by the momentum transfer that occurs in clouds of rising bubbles. The mass flow of air is constant for all the spargers used, at 100 L h^−1^. The ceramic sparger had a larger surface area than the limewood sparger and produced a dense cloud of smaller bubbles that increases the surface area of the bubble phase for momentum transfer within the liquid. As the surface area increases with a decrease in bubble size, and an increase in bubble number, more ambient liquid is dragged into the bubble cloud when compared with the larger bubbles produced by the limewood sparger (Zimmerman et al. 2008, 2009). A strong liquid circulation is established when the ceramic sparger is used dragging the surrounding fluid upwards and increasing the bubble’s velocity.

The bubbles within the foam phase have restricted movement and therefore do not respond to the bubble rise velocities of the freely moving bubbles within the liquid pool. The fast rise velocity of the foam produced by the LCF setup may be due to the surface area of the sparger relative to the aperture area of the harvester foam column. The entire area of the limewood sparger was able to fit directly under the column; therefore, almost all of the air was able to flow undisturbed directly from the liquid pool into the foam and up the column. In contrast, the ceramic flat plate sparger had a surface area that exceeded the column aperture resulting in the airflow being disturbed during its flow up the column. This resulted in longer foam residence times and allowed greater opportunity for liquid drainage (Morgan and Wiesmann 2001; Coward et al. 2013).

The significant changes recorded in the foam rise velocity produced by the LCF sparger over the course of the run period were likely due to surfactant exhaustion within the liquid pool restricting foam formation, thereby reducing the driving pressure within the foam column. This was often evidenced by the presence of void fractions (gas volume fraction) within the column, particularly in the latter stages of the run.

### Algal harvest

Studies of the effect of bubble size on the attachment efficiency of particles (Ahmed and Jameson 1985; Yoon and Luttrell 1989) have concluded that, under similar conditions, smaller bubbles produce higher attachment efficiencies than larger bubbles, thereby improving biomass recovery. Yoon and Luttrell (1989) noted that for bubbles larger than 350 μm, attachment efficiency decreased with increasing bubble diameter. This corresponds with the findings of this study. It can therefore be concluded that the COF setup generates an advantageous distribution of bubble sizes within the harvesting unit. The small bubbles produced in the liquid pool increase the collision rate and attachment efficiency, and the large slow-moving bubbles in the foam phase increase foam residence and liquid drainage.

### Harvest economics

It is difficult to conclude which is the most economically beneficial harvesting method based solely on harvesting efficiency. Energy consumption and chemical costs must also be considered. From Table 1, it can be seen that the energy consumption of the flotation unit is significantly increased when an oscillating flow is induced; this is due to the increase in operational pressure of the unit. However, the ceramic sparger used in these trials required 2.7 times less pressure than the stainless steel membrane diffusers used by Hanotu et al. (2012), which operated at a pressure of 0.8 bar. Much like the COF setup used in this experimental work, Hanotu et al. (2012, 2013) used air bleed-off or channelling of the airflow into another unit to control the airflow going into the column; however, the actual airflow entering the diffusers has not been noted. Therefore, a direct comparison of the energy consumption of the stainless steel membrane diffusers under an oscillating airflow could not be conducted.

The total cost of harvesting by foam flotation, using 10 mg L^−1^ of CTAB, was similar for all system setups, at a calculated cost of US$ 0.07–0.08. CTAB had a greater influence on the total harvesting cost than the price of electricity, as 98 % of the total operational cost of foam flotation is associated to the price of CTAB. For this economic assessment, it was assumed that although high airflows are required to induce the oscillation, the air would not be bled off before entering the system, but rather used for a number of units working in parallel. There is limited information available concerning the minimum supply airflow required to induce the oscillation, as the oscillation characteristics are dictated by the geometric parameters of the oscillator itself (Yang et al. 2007). For example, for a plane-wall oscillator, the oscillation occurs when the flow rate of the fluid exceeds 12 L min^−1^. However, step-wall oscillators are able to operate from 5 to 20 L min^−1^ (Yang et al. 2005). Tesar et al. (2006) stated that the frequency of the oscillation is controlled primarily by the length of the feedback loop and the supply flow rate. The fluidic jet-deflection amplifier used in these tests to induce the oscillating flow has previously been used between 10 and 80 L min^−1^ (Tesar et al. 2006; Hanotu et al. 2012, 2013); therefore, it can be assumed the minimum operating flow for the fluidic jet-deflection amplifier to be close to 10 L min^−1^. Although 3,700 L h^−1^ was needed to induce an oscillating flow, each unit harvesting 1,000 L of algae only required 100 L h^−1^ at 0.3 bar. Further investigation into the minimum operating flow to supply the fluidic jet-deflection amplifier in this system is required to ensure that all airflow is utilised efficiently, which would be more achievable with flow rates of 10 L min^−1^.

Assuming the same operating variables are used combining dispersed air flotation with fluidic oscillators, chemical dosing and pH adjustments (Hanotu et al. 2012, 2013) to induce flocculation followed by flotation results in a costly harvesting process (minimum of US$ 0.915 per 1,000 L); again, the total cost of harvesting is dominated by the chemical costs. This operating cost is not feasible for high-volume low-value products such as biodiesel feedstock.

Overall, COF provides the most advantageous cost-benefit relationship (gaining the equivalent biomass concentration of 27.8 g L^−1^), due to the higher biomass recovery of this method compared with LCF and CCF. Additionally, there remains scope to further optimise the COF method with respect to reducing the disturbance of the airflow into the foam phase, and with the addition of plates in the column to reduce liquid holdup within the foam (Li et al. 2011). Therefore, a COF sparger setup within a foam flotation harvesting system has the potential to reduce some of the financial barriers associated with many commonly used bulk microalgae harvesting techniques.

## Conclusion

Foam flotation is proving to be a viable and attractive method for harvesting microalgae biomass. The mechanism of bubble formation is a key design feature that significantly impacts on harvest efficiency as a function of bubble size and rise velocity. Oscillating airflow delivered the most effective biomass concentration results, and although the system had additional energy requirements necessary to attain an oscillating flow, the total harvesting costs (energy + chemical additives) make this approach attractive if growing microalgae for high-volume low-value products (biofuels). Combining CTAB-assisted foam flotation with the miniaturisation of bubbles within the liquid pool requires significantly lower rates of chemical addition compared with flocculation followed by dispersed air flotation.
